# Metabolic shifts in the Antarctic fish *Notothenia rossii* in response to rising temperature and *P*CO_2_

**DOI:** 10.1186/1742-9994-9-28

**Published:** 2012-10-18

**Authors:** Anneli Strobel, Swaantje Bennecke, Elettra Leo, Katja Mintenbeck, Hans O Pörtner, Felix C Mark

**Affiliations:** 1 Alfred Wegener Institute for Polar and Marine Research, Integrative Ecophysiology, Am Handelshafen 12 D-27570 Bremerhaven, Germany; 2 University of Trieste, Department of Life Science, via Giorgieri 1, Trieste, Italy

**Keywords:** Notothenioid, Oxygen consumption, Routine metabolic rate, Extracellular pH (pH_e_), Intracellular pH (pH_i_), Mitochondrial respiration, Acclimation, Acid–base

## Abstract

**Introduction:**

Ongoing ocean warming and acidification increasingly affect marine ecosystems, in particular around the Antarctic Peninsula. Yet little is known about the capability of Antarctic notothenioid fish to cope with rising temperature in acidifying seawater. While the whole animal level is expected to be more sensitive towards hypercapnia and temperature, the basis of thermal tolerance is set at the cellular level, with a putative key role for mitochondria. This study therefore investigates the physiological responses of the Antarctic *Notothenia rossii* after long-term acclimation to increased temperatures (7°C) and elevated *P*CO_2_ (0.2 kPa CO_2_) at different levels of physiological organisation.

**Results:**

For an integrated picture, we analysed the acclimation capacities of *N. rossii* by measuring routine metabolic rate (RMR), mitochondrial capacities (state III respiration) as well as intra- and extracellular acid–base status during acute thermal challenges and after long-term acclimation to changing temperature and hypercapnia. RMR was partially compensated during warm- acclimation (decreased below the rate observed after acute warming), while elevated *P*CO_2_ had no effect on cold or warm acclimated RMR*.* Mitochondrial state III respiration was unaffected by temperature acclimation but depressed in cold and warm hypercapnia-acclimated fish. In both cold- and warm-exposed *N. rossii*, hypercapnia acclimation resulted in a shift of extracellular pH (pH_e_) towards more alkaline values. A similar overcompensation was visible in muscle intracellular pH (pH_i_). pH_i_ in liver displayed a slight acidosis after warm normo- or hypercapnia acclimation, nevertheless, long-term exposure to higher *P*CO_2_ was compensated for by intracellular bicarbonate accumulation.

**Conclusion:**

The partial warm compensation in whole animal metabolic rate indicates beginning limitations in tissue oxygen supply after warm-acclimation of *N. rossii*. Compensatory mechanisms of the reduced mitochondrial capacities under chronic hypercapnia may include a new metabolic equilibrium to meet the elevated energy demand for acid–base regulation. New set points of acid–base regulation under hypercapnia, visible at the systemic and intracellular level, indicate that *N. rossii* can at least in part acclimate to ocean warming and acidification. It remains open whether the reduced capacities of mitochondrial energy metabolism are adaptive or would impair population fitness over longer timescales under chronically elevated temperature and *P*CO_2_.

## Introduction

Recent studies have demonstrated warming of the worlds’ oceans, and the Antarctic Peninsula also experiences a continuous increase in temperature [1-6]. Additionally, anthropogenic CO_2_-emissions accumulate in the atmosphere and the oceans [7] and result in a decrease in seawater pH (ocean acidification) [8-10]. Both ocean warming and acidification exert their specific effects on the marine fauna. Studies identifying the capacity of Antarctic fish to cope with thermal challenges [11-17] contributed to the concept of oxygen and capacity limited thermal tolerance, which explains the limits of thermal tolerance through limitations in tissue functional capacity and the associated oxygen limitation at high and low temperatures [13,18,19].

Changes in seawater temperature particularly affect cold stenothermal organisms, which generally possess extremely slow metabolic rates and poor acclimation capacities, when compared to temperate species [20,21]. While temperate ectothermic vertebrate and invertebrate species are apparently capable of shifting their temperature limits by acclimation [22], this feature and the potential interaction between effects of warming and increasing ocean acidification have been insufficiently explored in Antarctic fauna [11,23-25]. As stenothermal Antarctic fish are assumed to perform well only within their narrow environmental temperature range [15,26], the question arises whether these species can acclimate to increasing temperature and rising ocean *P*CO_2_.

Whole animal oxygen consumption rates reflect the energy demand of the organism as the sum of all physiological costs, including ion and acid–base regulation [20]. Under changing abiotic conditions, the rate of all or some of these processes may change, causing an increased or decreased overall demand for ATP. Therefore, the functional properties of mitochondria as the sites of energy (ATP) production may play a key role in shaping whole organism thermal tolerance and limits of aerobic metabolism [13,27,28]. However, the studies on mitochondrial respiration and capacities of Antarctic invertebrates [29,30] and fish [12,28,31-33] have so far not addressed changes in mitochondrial respiration after long-term acclimation to increased temperature or *P*CO_2_.

Increasing seawater *P*CO_2_ levels are hypothesized to narrow an organism’s thermal windows, possibly by limiting the ability to compensate for changes in acid–base status at thermal extremes [34-36]. Changes in acid–base status affect whole organism, cellular and molecular functions. Findings in teleost fish range from high compensatory abilities for rising seawater *P*CO_2_[37-39], to incomplete compensation with a long-term reduction in pH_e_[40].

Less is known about the response of intracellular pH (pH_i_) to changing temperature or hypercapnia in both marine vertebrates and invertebrates [41-44]. The intracellular proton buffering capacity of vertebrates is found to vary markedly between animal species, tissue type and aerobic capacity [45-48]. Especially studies on intracellular acid–base status in Antarctic fish are scarce [49].

The Antarctic fish species *N. rossii* is an abundant member of coastal Antarctic communities [50-52] and is widely distributed between 45° and ~ 62°S [53,54]. Water temperatures around the Antarctic Peninsula, e.g. in Potter Cove at King George Island, range from −2°C in winter to 2°C in summer [55]. *N. rossii* is adapted to this narrow thermal range and may display a limited resistance and acclimation capacity to warming compared to more eurythermal fish species.

The aim of this paper is to investigate the acclimation capacities of relevant components in aerobic metabolism of the Antarctic notothenioid *N. rossii* at increased seawater temperature and *P*CO_2_*.* In an integrative approach, we investigated acclimation to warming (7°C) and hypercapnia (0.2 kPa CO_2_) at different organisational levels, the whole animal, extracellular (blood) and intracellular, and the mitochondrial level. We exposed the animals to various abiotic conditions and then focused on changes in the fish’s condition and haematological parameters, and on oxygen consumption as a measure of routine metabolic rate (acute and after long-term acclimation). As a next step, we analysed mitochondrial acclimation and adaptation capacities (mitochondrial state III respiration, cytochrome c oxidase activity) as indicators of the plasticity of whole animal metabolic rate. Finally, we determined extra- and intracellular acid–base parameters in *N. rossii* as a measure of acid–base regulation patterns and capacities, and related them to the findings of reduced mitochondrial capacities under hypercapnia.

## Material and methods

### Animal capture and acclimation

Demersal marbled rockcod, *N. rossii*, were caught in December 2009 in Potter Cove, King George Island, Antarctic Peninsula (62°14’S; 058°41’W) during the Antarctic summer season (seawater temperature 0.8°±0.9°C, salinity 33.5±0.2 psu). Fish were collected using baited traps (length 124 cm, width 64 cm, height 56 cm, mesh size 25 mm) and trammel nets (length 15 m, inner mesh 25 mm).

Animals were reared and acclimated in the aquaria facilities at Dallmann Laboratory, Carlini Station (formerly Jubany Station, King George Island) with direct seawater supply from the cove, under natural light conditions. Following the Intergovernmental Panel on Climate Change’s “business-as-usual” scenario, atmospheric CO_2_-concentrations may exceed 0.2 kPa by the year 2200 [8,56]. Therefore, we chose 0.2 kPa CO_2_ for our hypercapnia acclimation of *N. rossii*. For acclimation, animals were randomly selected and acclimated to 1°C, 0.04 kPa CO_2_ (control group, *n*=9, mass 155–804 g; total length 25–39.4 cm), 1°C, 0.2 kPa CO_2_ (cold hypercapnic group, *n*=10, mass 144–510 g; total length 23.8-32.8 cm), 7°C, 0.04 kPa CO_2_ (warm normocapnic group, *n*=5, mass 151–412 g; total length 23.6-33.9 cm) and 7°C, 0.2 kPa CO_2_ (warm hypercapnic group, *n*=10, mass 137–504 g; total length 21.4-31.3 cm). Animals were fed to satiation twice per week with chopped fish muscle and snails.

For all acclimations, seawater temperature (from 1° to 7°C) and *P*CO_2_ (from 0.04 kPa CO_2_ to 0.2 kPa CO_2_) were both increased stepwise (1°C/4 hours; 0.01 kPa CO_2_/h) over 24 hours. Total acclimation time was 29–36 days. Experimental animals were acclimated in well-aerated (>95% O_2_ saturation), 150 liter tanks, fed by an additional 150 liter header tank. This header tank was used for a daily water exchange of 150 liter to avoid alteration of the conditions in the acclimation tanks. For the warm normocapnia/ hypercapnia acclimations, temperature was kept constant using a 250 W heating element (Jaeger, EHEIM GmbH, Germany), controlled by a Temperature Controller TMP1380 (iSiTEC GmbH, Germany). For the cold/ warm hypercapnia acclimations, higher *P*CO_2_ was regulated by an iks aquastar system (iks ComputerSysteme GmbH, Germany). The system maintained constant pH (accuracy ± 0.05 pH units) by controlling a solenoid valve (Aqua Medic GmbH, Germany), which bubbles the acclimation tanks with pure CO_2_. Specific seawater conditions are given in Table 1. pH of all acclimation systems was measured daily with a WTW 340i pH meter (WTW, Germany. Electrode: WTW SenTix HWS) and calibrated daily with NBS (WTW, Germany) buffer. Total CO_2_ (C_CO2_) in the seawater was determined with a carbon dioxide analyser (Corning 965, CIBA, Corning Diagnostics, England). Seawater carbonate chemistry was calculated with the measured pH_NBS_ and C_CO2_ using the CO2sys software [57]. All experiments were conducted at Dallmann Laboratory (Carlini Station), King George Island, Antarctic Peninsula.

**Table 1:** **Seawater physiochemical conditions of the control conditions and different warm/ hypercapnia acclimations of *N. rossii *at Carlini station**

**Conditions**	**Control**	**Warm normocapnia**	**Cold hypercapnia**	**Warm hypercapnia**
	**(1°C, 0.04 kPa CO**_**2**_**)**	**(7°C, 0.04 kPa CO**_**2**_**)**	**(1°C, 0.2 kPa CO**_**2**_**)**	**(7°C, 0.2 kPa CO**_**2**_**)**
pH	8.250±0.015	7.914±0.016	7.455±0.018	7.493±0.006
*P*_CO2_ (μatm)	356.72±18.26	461.43±13.69	2179.04±54.50	2018.55±26.93
*P*_CO2_ (kPa)	0.037±0.002	0.048±0.001	0.225±0.006	0.208±0.003
DIC (mmol/kgSW)	2595.00±118.66	1942.50±26.39	2401.81±5.58	2369.44±2.04
HCO_3_- (mmol/kgSW)	2429.78±110.06	1839.93±26.00	2254.02±3.54	2241.68±1.06
T (°C)	1.45±0.09	7.25±0.07	1.36±0.17	6.88±0.063
S (psu)	32.47±0.35	32.75±0.21	32.77±0.29	32.63±0.26
duration (weeks)	1	4	4	4

### Routine metabolic rate

Routine metabolic rate (RMR) of *N. rossii* (control/ after acute temperature elevation/ long-term acclimated) was measured via intermittent-flow respirometry. Following Johnston et al. [58], fish were not fed for 10 days prior to the respiration experiments. After the acclimation period, each fish was placed in a 3500 to 4400 ml non-transparent, cylindrical respirometer placed within a 150 liter tank under acclimation conditions. Individuals were allowed to recover within the respiration chamber for 24 hours, a period considered appropriate to overcome the effect of any handling stress [58]. A constant, circulating water flow in the respirometer was generated by an aquarium pump. In the intermittent-flow system, water exchange between chamber and ambient water was interrupted every 30 min for 15 or 30 min to measure oxygen depletion (max. 10% O_2_) by the fish within the chamber, then oxygen concentration was replenished to 100% by flush pumps. Oxygen concentration within the chamber was detected once per minute using a FiBox2 (PreSens – Precision Sensing GmbH, Germany) oxygen meter. The device was calibrated before each measurement in well-aerated seawater at the respective acclimation temperature, calibration at zero oxygen was conducted in nitrogen-bubbled seawater.

In three individuals of *N. rossii,* oxygen consumption was measured before and after acute temperature increase. The same experimental setup as described above was used. After 24 hours of recovery, RMR was recorded for 24 hours under control conditions (1°C), then temperature was increased continuously by 1°C per hour up to 7°C. RMR was recorded at the beginning and at the end (7°C) of the acute warming period. Mean RMR were calculated over 24 hours, and thus represent resting metabolism including spontaneous activity. Blank measurements of bacterial respiration in the respirometer were carried out for each acclimation group, values for RMR were corrected accordingly.

### Animal condition, sampling and haematological parameters

After measurement of RMR and at the end of the acclimation period, specimens of *N. rossii* were anesthesized with 0.5 g/l tricaine methano- sulphonate (MS 222). Blood was taken with a syringe from the caudal vein, the liver was taken for mitochondrial isolation. Parts of liver and muscle samples were immediately freeze-clamped and frozen in liquid nitrogen for pH_i_ analysis, as described by Pörtner [47]. Afterwards, individuals were killed by a spinal cut behind the head plates. The work was carried out according to the ethics and guidelines of German law. Experiments had been approved according to § 8 animal welfare act (18.05.2006; 8081. I p. 1207) by the veterinary inspection office, Bahnhofsplatz 29, 28195 Bremen, Germany, under the permit number Az.: 522-27-11/02-00 (93) on January 15th, 2008 (permit valid until Jan 14th 2012).

Fulton’s condition factor (CF) of *N. rossii* was calculated according to the formula [59]:

(1) $$\text{CF}=\text{animal weight}\;\left[\text{g}\right]\times 100/\text{standard length}\;{\left[\text{cm}\right]}^{3}$$

The hepatosomatic index (HSI) was determined by

(2) $$\text{HSI}=\text{liver weight}\;\left[\text{g}\right]/\text{total weight}\;\left[\text{g}\right]\times 100$$

The haematocrit of *N. rossii* was estimated in a blood subsample using a haematocrit centrifuge (Compur Microspin, Bayer Diagnostics Mfg. Ltd., Microspin, Ireland). Lactate concentration was measured with an Accutrend® Lactate tester (Roche Diagnostics GmbH, Germany). Osmolarity of the serum was measured after centrifugation of the blood for 10 min at 2000 g. For the measurement, a Vapour Pressure Osmometer 5500 (Wescor Inc., USA) was used.

### Isolation of liver mitochondria and mitochondrial oxygen consumption measurements

Immediately after excision, the liver was rinsed and total liver weight was determined before a subsample of liver tissue was taken, weighed and rinsed with 5 ml/g ice-cold isolation buffer containing 80 mM sucrose, 85 mM KCl, 5 mM EGTA, 5 mM EDTA, 50 mM HEPES and 1% w/v bovine serum albumin (BSA, fatty acid free) (pH 7.1 at 20°C). The liver tissue was then finely minced with scissors, suspended in 10 volumes ice-cold isolation buffer, and then put into a 30 ml Potter-Elvehjem glass homogenizer (VWR International, Germany) and slowly homogenised with three strokes at 80 revolutions/ minute. The homogenate was centrifuged (600 g, 10 min., 0°C), the supernatant collected and the pellet vigorously resuspended by vortexing in isolation buffer and centrifuged for a second time. Supernatants were then combined and centrifuged for 10 min at 11.000 g (0°C). The supernatant was discarded, any remaining droplets of fat removed with a cotton swab and the pellet resuspended in isolation buffer and centrifuged again. As a last step, supernatant was discarded again, and the pellet was resuspended in ice-cold mitochondria assay buffer (80 mM sucrose, 85 mM KCl, 5 mM KH_2_PO_4_, 50 mM HEPES, 1% w/v BSA (fatty acid free), pH 7,1 at 20°C) at 1 ml/g initial liver weight. This mitochondrial preparation was kept on ice away from light and used for mitochondrial oxygen consumption measurements. The mitochondrial protein concentration was determined according to Bradford [60] using a bovine serum albumin (BSA) standard, and considering the protein content of the mitochondrial assay buffer.

Mitochondrial respiration measurements were conducted in two thermostatted perspex respiration chambers of 3 ml volume (World Precision Instruments, Inc., USA), equipped with an adjustable stopper and ports for the injection of metabolites and inhibitors and one for insertion of a TX micro-optode (PreSens – Precision Sensing GmbH, Germany), used for fluoroptic measurement of *P*O_2_. The oxygen traces were recorded with a PowerLab recording unit and Chart v5.5.6 software (ADInstruments GmbH, Germany). Mitochondrial respiration rates were converted to nmol O_2_*mg extracted mitochondrial protein^-1^*min^-1^.

Measurements were carried out in assay buffer with a final volume of 1200 μl with mitochondrial concentrations adjusted to about 3 mg mitochondrial protein per ml, at 0, 6, and 12±0.1°C, respectively. Chamber temperature was maintained with a thermostat (LAUDA, Germany). Respiration was recorded and malate and pyruvate added to a final concentration of 1.3 mM and 1.6 mM, respectively, as substrates for complex I (state II), and ADP (final conc. 0.16 mM) was added to measure state III (max. slope). Then, 2 mM succinate was added as complex II substrate and 0.16 mM ADP for state III respiration.

### Enzyme assays

Frozen liver tissue was ground into powder by mortar and pestle under liquid nitrogen and homogenized in a glass homogenizer in 9 vol. buffer containing 20 mmol l^-1^ Tris–HCl, 1 mmol l^-1^ EDTA, 0.1% Triton X-100, pH 7.4, and afterwards with an Ultra Turrax (Silent Crusher M (Heidolph Instruments, Germany), followed by 10 min centrifugation at 1,000 *g* at 4°C*.* Cytochrome *c* oxidase (COX) activity was determined according to a protocol modified from Moyes et al. [61] in buffer containing 20 mmol l^-1^ Tris–HCl, 0.05% Tween 20 and 0.057 mM reduced cytochrome *c* at pH 8.0. The decrease in extinction at λ = 550 nm through oxidation of cytochrome *c* (ε_550_ = 19.1 mol^-1^cm^2^) was monitored in a thermostatted spectrophotometer (Beckman, Fullerton, CA, USA) at 0, 6 and 12°C. Protein concentration of the tissue extract was determined according to Bradford [60], enzyme activity is given in μmol*mg protein^-1^*min^-1^.

### Acid–base parameters

#### Intracellular acid–base variables

Measurement of pH_i_ was carried out according to the homogenization technique developed by Pörtner [47]. A solution of 1 mM nitrilotriacetic acid (NTA) and 160 mM potassium fluoride (KF) was used to keep the NTA concentration as low as possible. Cco_2_ was measured by gas chromatography (6890N Network GC System, Agilent Technologies), total CO_2_ in cell water was calculated according to Pörtner [47], assuming a fractional tissue water content of 0.78 [62]. Intracellular acid–base parameters were calculated using the following, modified Henderson-Hasselbalch equation.

(3) $$P{\text{CO}}_{2}={{\text{C}}_{\text{CO}}}_{2}\text{x}\;{\left(10^{\text{pH}-\text{pK}\prime \prime \prime }\text{x}\;\alpha +\alpha \right)}^{-1}$$

(4) $$\left[{{\text{HCO}}_{3}}^{-}\right]={\text{Cco}}_{2}-\alpha P{\text{CO}}_{2}$$

Intracellular pK”’ and α (solubility) values were evaluated according to Heisler [62] using [Na^+^ = 0.02 M, [M] = 0.21 mol l^-1^, I = 0.12 mol l^-1^ and [Protein] = 220 g l^-1^[47].

The tissue buffer values β_NB_ for liver and muscle were adopted from Van Dijk et al. [63].

#### Extracellular values

Blood plasma pH (extracellular pH, pH_e_) was measured immediately after sampling at the acclimation temperature with a pH meter (WTW 340i, WTW, Germany. Electrode: InLab® Viscous, Mettler Toledo GmbH, Germany). The pH meter was calibrated daily with NBS buffers (WTW, Germany). Measurements were carried out in a closed microcentrifuge tube (0.5 ml) to minimize contact with environmental air. Plasma total CO_2_ (C_CO2_) was measured after centrifugation by means of a carbon dioxide analyser (Corning 965, CIBA, Corning Diagnostics, England). Blood carbonate chemistry was calculated using the modified Henderson-Hasselbalch equation (eqn. 3 and 4). Values for the CO_2_-solubility coefficient α and the negative logarithm of the dissociation constant K”’ were calculated after Heisler [62]. The values required for the calculation (ionic strength, protein concentration, Na^+^ concentration) were adopted from Egginton [64].

#### Blood non-bicarbonate buffer value (Î²_NB_)

After heparinization of the blood (100 U/ml), the β_NB_ capacity was determined at 0.5°C in a thermostatted tonometer. 1.5 ml of whole blood was equilibrated with different *P*CO_2_ (1 kPa, 2 kPa, 3 kPa CO_2_) for 1 hour before pH (electrode: InLab® Viscous, Mettler Toledo GmbH, Germany) and C_CO2_ were measured (gas chromatography: 6890N Network GC System, Agilent Technologies). The β_NB_ capacity (−Δ[HCO_3_^-^]/ΔpH) was determined with values for Δ[HCO_3_^-^] calculated after equation 4.

### Data analysis and statistics

The temperature coefficient Q_10_ was calculated for routine metabolic rate and mitochondrial respiration (state III) according to the formula

(5) $${\text{Q}}_{10}={\left({{\text{MO}}_{2}}_{\left(2\right)}/{{\text{MO}}_{2}}_{\left(1\right)}\right)}^{10/\left({\text{T}}_{2}-{\text{T}}_{1}\right)}$$

The respiratory control ratio (RCR) was calculated as the ratio between mitochondrial state III (complex I and II) and state IV (after ADP depletion) respiration.

All data were tested for outliers at the 95% significance level using Nalimov’s test [65] as well as for normality (Kolmogorov-Smirnov) and homogeneity of variance. Differences in routine metabolic rate, mitochondrial oxygen consumption and COX activity at the assay temperatures 0, 6, 12°C, blood and intracellular acid–base variables, between the different acclimation groups were tested using unpaired, two-tailed t-tests and one-way analysis of variance (ANOVA, with Tukey post-hoc test). *p≤*0.05 was considered the significance threshold. All data are presented as means ± standard error of the mean (SEM).

## Results

### Animal condition and haematological parameters

The relative liver weights (hepatosomatic indices, HSI), condition factors, haematocrits, blood osmolarities and lactate levels, as well as the blood acid–base parameters determined for control and acclimated *N. rossii*, are summarized in Table 2. The HSI showed a non-significant tendency to decrease after warm-acclimation of *N. rossii* in both the normocapnic (7°C, 0.04 kPa CO_2_) and the hypercapnic (7°C, 0.2 kPa CO_2_) groups. Acclimation to higher *P*CO_2_ at 1°C had no significant effect on the HSI. The condition factor of *N. rossii*, indicating nutritional status, was significantly decreased in the warm hypercapnic group. The haematocrit of *N. rossii* displayed no acclimation effect. Lactate concentrations in the blood, on average, remained below detection limit (<0.8 mmol*l^-1^). Only in the cold hypercapnic group, lactate was slightly elevated to 1.13±0.11 mmol*l^-1^. The blood osmolarity in *N. rossii* was significantly reduced in all warm and hypercapnia acclimated animals.

**Table 2:** **Animal condition (HSI= hepatosomatic index, CF=condition factor), and blood parameters of the Antarctic fish *N. rossii***

**Acclimation**							
**T**	***P*CO**_**2**_	**N**	**HSI**	**CF**	**Haematocrit**	**Lactate**	**Osmolarity**
(°C)	(kPa)					(mmol*l^-1^)	(mOsm*l^-1^)
1	0.04	9	1.83±0.33	1.69±0.03	28±2	<0.8	436.8±9.5
7	0.04	9	0.91±0.04^*^	1.57±0.07	29±2	<0.8	373.8±15.7^*^
1	0.2	5	1.22±0.12	1.59±0.03	29±1	1.13±0.11	399.5±13.3
7	0.2	10	0.81±0.06^*^	1.5±0.03^*^	31±1	<0.8	390.0±8.6^*^

Control: 1°C, 0.04 kPa CO_2_; warm normocapnia: 7°C, 0.04 kPa CO_2_; cold hypercapnia: 1°C, 0.2 kPa CO_2_; warm hypercapnia: 7°C, 0.2 kPa CO_2_*.*

All values are given as means ± SEM. * indicate a significant difference to control conditions (*t*-test, *p≤*0.05).

### Routine metabolic rate

**Figure 1:**
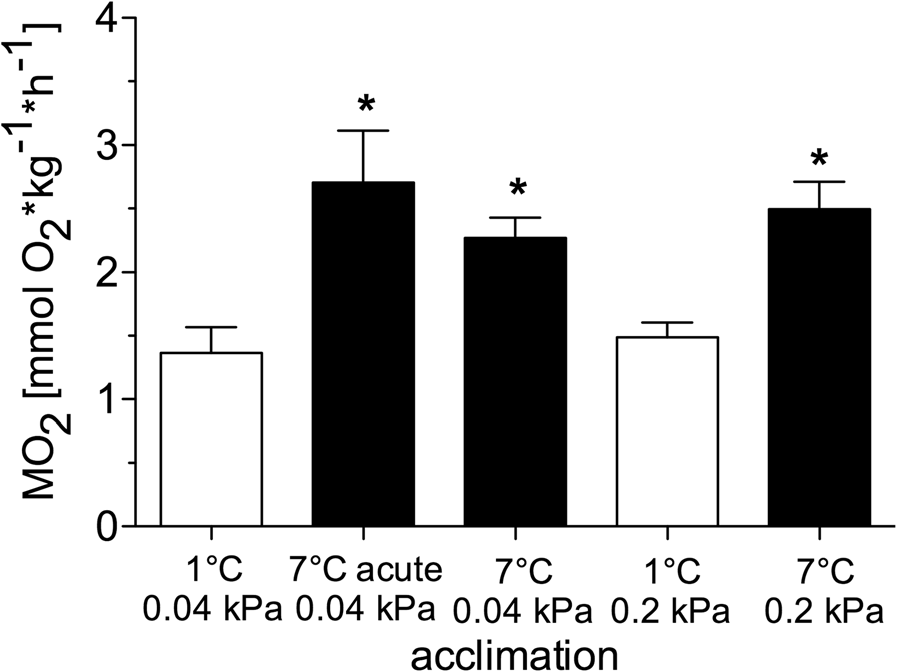
**Routine metabolic rate of *N. rossii *at different acclimation conditions.** Oxygen consumption of control, cold hypercapnia (white bars), warm normocapnia/ hypercapnia (black bars)) acclimated fish, and after acute warming (1°C/ hour) to 7°C, normocapnia. All data are presented as means ± SEM, *n=*3-6. * indicate significant difference of warm/ hypercapnia acclimation/ acute warming to control *N. rossii* (*t*-test, *p≤*0.05).

*N. rossii* exposed to acute warming (7°C) showed significantly increased, two-fold higher RMR compared to the control group (1.35±0.20 (control at 1°C, *n*=5) *vs.* 2.71±0.41 (acutely warmed to 7°C, *n*=3) mmolO_2_*kg^-1^*h^-1^), as displayed in Figure 1.

Oxygen consumption in the control group and the long-term warm and/ or hypercapnia acclimated *N. rossii* was measured at the end of the 4-weeks acclimation period. In comparison to the control group, the RMR of the long-term warm and normocapnia acclimated fish was significantly increased at 7°C (2.23±0.16 mmol O_2_*kg^-1^*h^-1^ (*n*=5), Q_10_ of 2.38). However, after long-term warm acclimation RMR was significantly lower compared to that of the acutely warmed *N. rossii* at 7°C (Q_10_ of 3.2). RMR after cold hypercapnia acclimation (1.28±0.10 mmol O_2_*kg^-1^*h^-1^, *n*=6) was similar to that in control animals. Warm hypercapnia acclimation resulted in a significantly increased RMR (2.49±0.22 mmol O_2_*kg^-1^*h^-1^, *n*=5, measured at 7°C) compared to the control group measured at 1°C. No significant difference in RMR was found between the long-term warm normocapnic and the warm hypercapnic groups.

### Mitochondrial respiration, respiratory control ratio (RCR) and mitochondrial Q_10_

**Figure 2:**
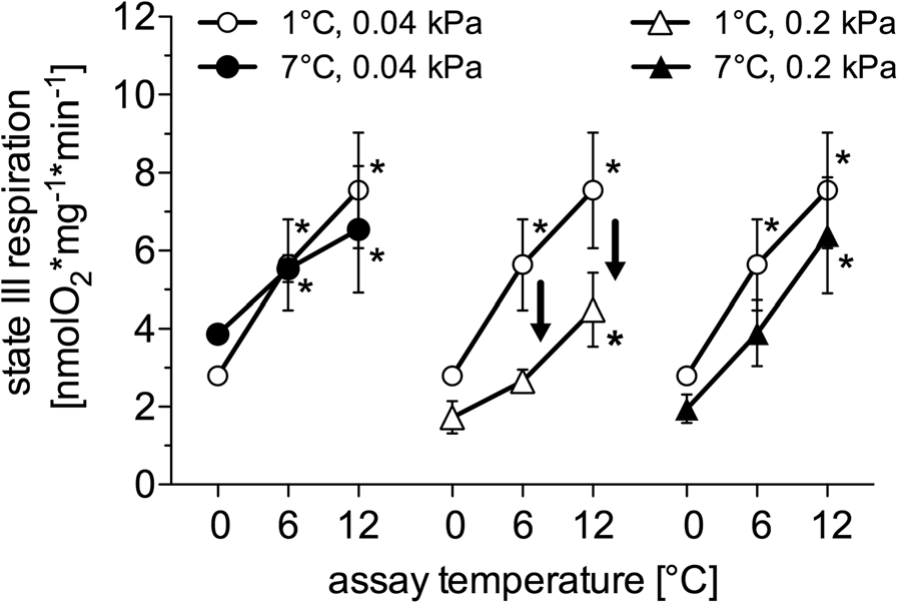
**State III respiration rate in liver mitochondria of *N. rossii*.** State 3 respiration was measured in the presence of malate, pyruvate, succinate and ADP at the respective assay temperature (0°C, 6°C, 12°C) in four different long-term acclimated groups; open circles: 1°C, 0.04 kPa CO_2_ (control); filled circles: 7°C, 0.04 kPa CO_2_ (warm normocapnic); open triangles: 1°C 0.2 kPa CO_2_ (cold hypercapnic); filled triangles: 7°C 0.2 kPa CO_2_ (warm hypercapnic).* indicate significantly higher mitochondrial respiration than at 0°C assay. ↓ depicts significantly reduced mitochondrial respiration in comparison to control acclimation (1°C, 0.04kPa CO_2_). All data are presented as means ± SEM, *n=*5-10.

Liver mitochondrial state III respiration increased significantly when assay temperature was changed acutely from 0°C (2.79±0.18 nmol O_2_*mg*min^-1^) to 6°C (5.64±1.17 nmol O_2_*mg*min^-1^) and 12°C (7.55±1.49 nmol O_2_*mg*min^-1^) in the control group (*n*=10), with similar rates in the warm normocapnic fish (*n*=5; 0°C: 3.86±0.19; 6°C: 5.53±0.34; 12°C: 6.55±1.62 nmol O_2_*mg*min^-1^). In cold hypercapnia (*n*=9; 0°C: 1.73±0.42; 6°C: 2.65±0.30; 12°C: 4.49±0.94 nmol O_2_*mg*min^-1^) and warm hypercapnia acclimated fish, state III respiration was significantly increased only at 12°C (*n*=9; 0°C: 1.95±0.36; 6°C: 3.89±0.85; 12°C: 6.40±1.50 nmol O_2_*mg*min^-1^) (Figure 2). In the cold hypercapnic animals, state III respiration was significantly reduced (at 6°C and 12°C in the assay) compared to the control group.

Within each experimental group, RCR did not vary significantly between the different assay temperatures (0, 6, 12°C), whereas between the experimental groups the mean RCR of the 0, 6 and 12°C assays were significantly reduced in both cold and warm hypercapnia acclimated animals compared to the control group (Table 3). There was no difference in the Q_10_ values for mitochondrial state III respiration in the acute assay temperature range from 0-12°C between the different acclimation groups.

**Table 3:** **Respiratory control ratio (RCR; for each assay temperature and as mean RCR over all three assay temperatures at the respective acclimation) and Q**_**10**_**of *N. rossii *(control: 1°C, 0.04 kPa CO**_**2**_**; warm normocapnia: 7°C, 0.04 kPa CO**_**2**_**; cold hypercapnia: 1°C, 0.2 kPa CO**_**2**_**; warm hypercapnia: 7°C, 0.2 kPa CO**_**2**_**)**

**Acclimation**			**RCR**		**Q**_**10**_	
**Temperature**	***P*CO**_**2**_	**Assay temp.**	**CI+CII**		**0-12°C**	
**[°C]**	**[kPa]**	**[°C]**		**mean**		**N**
1	0.04	0	4.9±0,4	4.6±0.8	1.6±0.3	5
		6	5.2±0.8			5
		12	3.6±0.2			5
7	0.04	0	5.0±0.6	4.0±1.0	1.8±0.3	5
		6	3.9±0.6			5
		12	3.0±0.9			5
1	0.2	0	4.6±0.6	3.8±0.8*	2.4±0.4	10
		6	4.0±0.6			10
		12	3.0±0.4			10
7	0.2	0	4.2±0.6	3.5±0.6*	2.1±0.3	9
		6	3.4±0.3			9
		12	2.9±0.2			9

* indicates a significantly different RCR compared to control *N. rossi*.

### Enzyme activities

**Figure 3:**
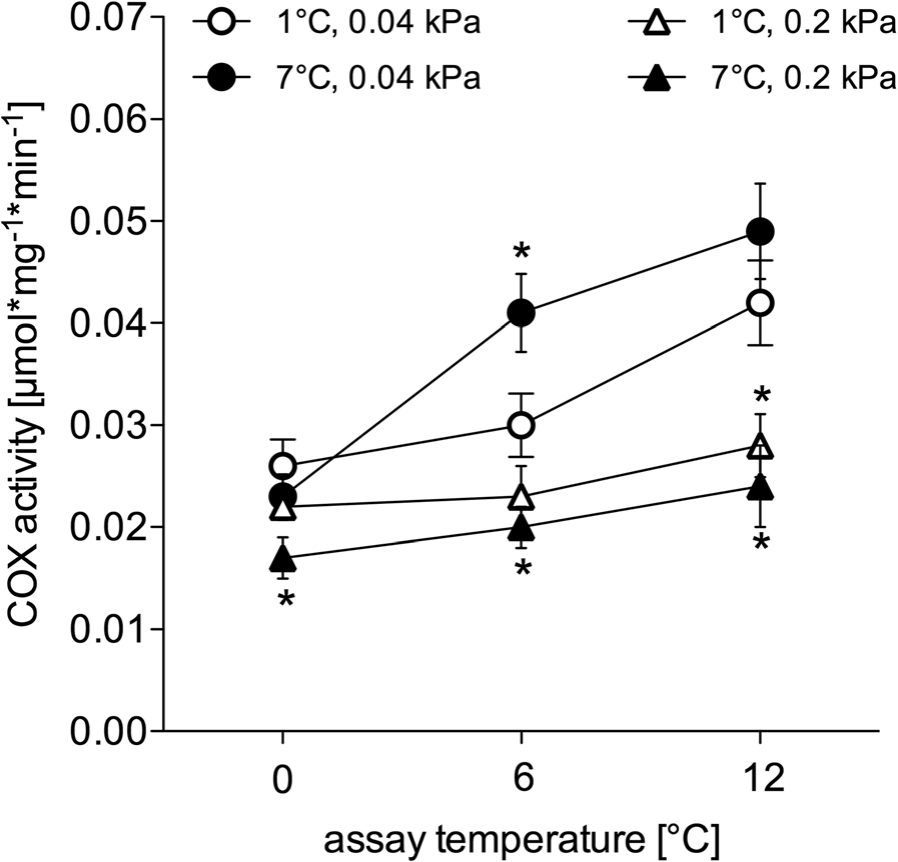
**Cytochrome *c *oxidase (COX) activity in liver extracts of long-term acclimated *N. rossii*.** Open circles: 1°C, 0.04 kPa CO_2_ (control); filled circles: 7°C, 0.04 kPa CO_2_ (warm normocapnic); open triangles: 1°C 0.2 kPa CO_2_ (cold hypercapnic); filled triangles: 7°C 0.2 kPa CO_2_ (warm hypercapnic). * depicts a significantly higher/ lower enzyme activity of the respective acclimation group compared to control, at the given assay temperature. Activity values are given as means ± SEM in μmol*mg^-1^*min^-1^, *n=*4-7.

In the warm hypercapnic group, COX activities in liver were decreased compared to the control at all temperatures. A similar trend was apparent in the cold hypercapnic animals, but only significant in the assay at 12°C (Figure 3). COX activities in liver extracts of the warm normocapnic group were significantly higher than in the control group at the 6°C assay.

### Acid–base parameters of N. rossii

#### Intracellular

The pH_i_ of muscle tissue of the control *N. rossii* was 7.33±0.07 (*n*=3) (Table 4). The highest, though not significant deviation from the control group was found in the warm normocapnic animals (0.08 pH units lower). Liver pH_i_ was lower than the respective muscle pH_i_ in all treatments, control pH_i_ of liver tissue was 7.08±0.03 (*n*=3). In parallel, liver *P*CO_2_ in all acclimation groups was significantly higher than muscle *P*CO_2_. All acclimation groups showed muscle tissue [HCO_3_^-^] elevated above controls ([HCO_3_^-^] = 3.99±0.41 mM), up to 6.72±0.66 mM (*n*=5) in cold hypercapnic specimens and 6.85±0.37 mM (*n*=10) in warm hypercapnic individuals.

**Table 4:** **Intracellular and extracellular pH (pH**_**i/ e**_**) and acid–base parameters in white muscle/ liver tissue homogenates and blood, respectively, from *N. rossii*. Control: 1°C, 0.04 kPa CO**_**2**_**; warm normocapnia: 7°C, 0.04 kPa CO**_**2**_**; cold hypercapnia: 1°C, 0.2 kPa CO**_**2**_**; warm hypercapnia: 7°C, 0.2 kPa CO**_**2**_

**Acclimation**		**White muscle homogenate**				**Liver homogenate**				**Blood acid–base parameters**			
**T (°C)**	***P*CO**_**2 **_**(kPa)**	**N**	***P*CO**_**2 **_**(kPa)**	**[HCO**_**3**_^**-**^] (mmol*l^-1^)	**pHi**	**N**	***P*CO**_**2 **_**(kPa)**	**[HCO**_**3**_^**-**^](mmol*l^-1^)	**pHi**	**N**	***P*CO**_**2 **_**(kPa)**	**[HCO**_**3**_^**-**^] (mmol*l^-1^)	**pHe**
1	0.04	3	0.640±0.126	3.994±0.410	7.325±0.065	3	2.555±0.270	9.213±0.379	7.080±0.027	9	1.18±0.19	8.05±0.56	7.438±0.055
7	0.04	5	1.071±0.123	5.294±0.335	7.242±0.037	1	3.616	8.020	6.890	9	1.16±0.05	6.31±0.28^a,b^	7.315±0.045^b^
1	0.2	5	0.936±0.117	6.724±0.664^a^	7.383±0.027	5	3.031±0.484	10.107±1.197	7.053±0.066	5	1.46±0.09	11.28±0.32^a,b,c^	7.508±0.028
7	0.2	10	1.296±0.167	6.849±0.373^a^	7.290±0.038	7	4.495±0.521	10.347±0.724	6.920±0.039	10	1.41±0.11	10.08±0.57^a^	7.507±0.037

Data are presented as means ± SEM. Superscript letters indicate following significant differences: a – sign. different to control conditions, b – sign. different to temperature & CO_2_ acclimation. c –sign. different to temperature acclimation (*t*-test, ANOVA, *p≤*0.05).

Liver [HCO_3_^-^] was generally higher than in muscle samples. [HCO_3_^-^] in liver under control conditions (9.21±0.37 mM, *n*=3) was slightly lower than in the cold hypercapnic group (10.11±1.19 mM, *n*=5) and the warm hypercapnic group (10.35±0.72 mM, *n*=7). These data have to be considered with caution, as due to sample shortage (most liver tissue was needed for isolation of mitochondria) there was only one liver sample available for pH_i_ estimation in the warm normocapnic group, however, with a value similar to those in the warm hypercapnic group (*n*=7).

**Figure 4:**
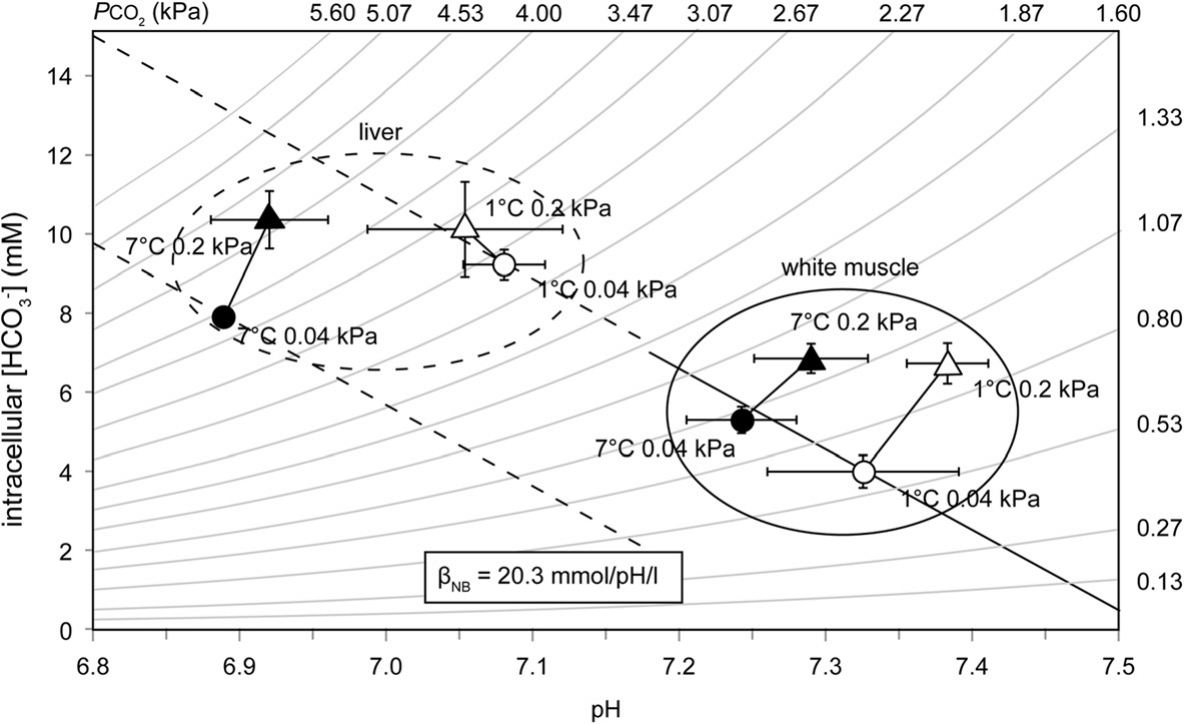
**pH**_**i**_**/ [HCO**_**3**_^**-**^**] (Davenport) diagram of intracellular pH (pH**_**i**_**) and acid–base parameters.** Values of liver and white muscle homogenates of control (1°C, 0.04 kPa CO_2_) and warm normocapnia (7°C 0.04 kPa CO_2_), cold hypercapnia (1°C, 0.2 kPa CO_2_) and warm hypercapnia (7°C, 0.2 kPa CO_2_) acclimated *N. rossii* (c.f. Table 4). All data are given as means ± SEM, *n=*1-7. The solid line marks the non-bicarbonate buffer line (β_NB_) for white muscle, the dashed line for liver. β_NB_ value was adopted from VanDijk et al. [63] for the Antarctic eelpout *P. brachycephalum*.

Visualisation of the intracellular acid–base parameters in a pH-bicarbonate diagram (Figure 4) illustrates that in liver tissue, changes in acid–base status of the cold hypercapnic group changed in parallel to the assumed non-bicarbonate buffer line of controls; only in the warm normocapnic *N. rossii* was the resulting value located distinctly below the non-bicarbonate buffer line of controls. In white muscle, the CO_2_ induced acidosis in both cold and warm hypercapnic groups was compensated for by a significant rise in [HCO_3_^-^], resulting in values close to the *P*CO_2_ isobar of ~ 0.95 and 1.15 kPa CO_2_, respectively.

#### Extracellular

**Figure 5:**
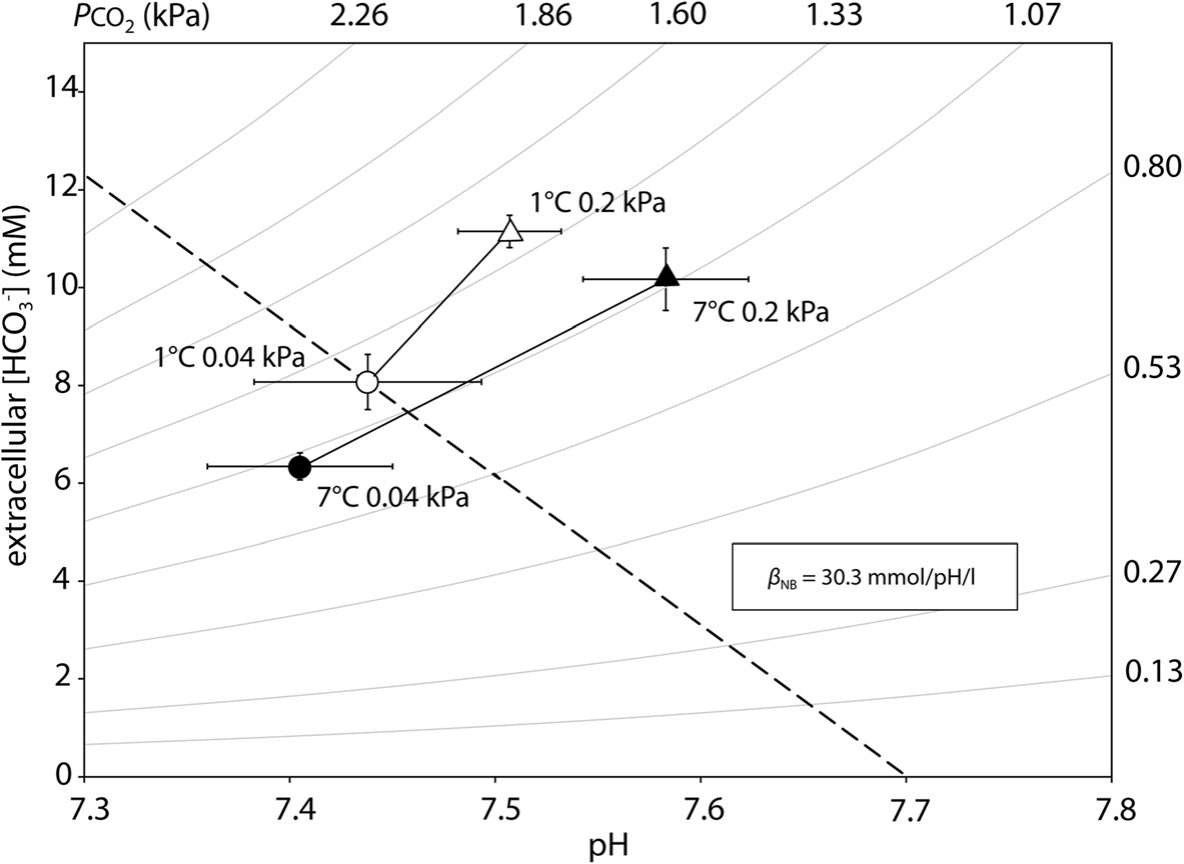
**pH**_**e**_**/ [HCO**_**3**_^**-**^**] (Davenport) diagram of the extracellular acid–base variables.** Acid–base parameters are depicted for control (1°C 0.04 kPa CO_2_) and warm normocapnia (7°C 0.04 kPa CO_2_), cold hypercapnia (1°C, 0.2 kPa CO_2_) and warm hypercapnia (7°C, 0.2 kPa CO_2_) acclimated *N. rossii* (c.f. Table 4). The dashed line marks the *in vitro* non-bicarbonate buffer line (β_NB_), curved lines represent *P*CO_2_ isopleths. All data are presented as means ± SEM, *n=*5-10.

Cold hypercapnia acclimation did not significantly affect pH_e_, which was 7.44±0.06 (*n*=9) under control conditions (Table 4). pH_e_ was significantly higher in the warm hypercapnic group (7.51±0.04, *n*=10) than in the warm normocapnic (7.32±0.05, *n*=9) group. None of the acclimations significantly affected extracellular *P*CO_2_, however, changes in bicarbonate result as a consequence of CO_2_ enrichment from 1.2 to 1.4 kPa in warm and from 1.2 to 1.5 kPa in cold acclimated animals. Cold hypercapnia acclimation was in fact associated with a significant increase in bicarbonate levels ([HCO_3_^-^] control: 8.05 mmol*l^-1^, cold hypercapnic 11.28 mmol*l^-1^). In contrast, blood [HCO_3_^-^] of the warm normocapnic animals was significantly decreased (6.31 mmol*l^-1^). When displayed in a pH-bicarbonate diagram (Figure 5), together with the blood buffer line β_NB_ of 30.3 mmol*pH^-1^*l^-1^ which we measured for *N. rossii* at 0°C, it becomes obvious that long-term hypercapnia acclimation involves a marked accumulation of [HCO_3_^-^], resulting in elevated steady state levels.

## Discussion

### Aerobic energy metabolism

During acute warming, the RMR of *N. rossii* increased as a result of rising metabolic rate with a Q_10_ of 3.1 (Figure 1), which is consistent with previous studies on acute thermal responses of different Antarctic fish species [11,26,66,67]. A part of the increase in metabolic rate can likely be attributed to the high costs of the low capacity cardio-vascular system to meet the increasing metabolic oxygen demand, which has been demonstrated for Antarctic eelpouts [13,68]. Due to limited cardiac scope and increased friction of the vascular system at high blood flow rates, sufficient oxygen delivery at warm temperatures result in a relatively higher workload for the heart. A compensation of cardiac scope during long-term acclimation may alleviate this to some extent, contributing to a lower RMR and Q_10_ of 2.3 after warm acclimation (Figure 1), which indicate a partial, incomplete compensation of RMR in *N. rossii* (type 3 after Precht [69]). The same effect occurred in the long-term warm hypercapnic acclimated *N. rossii*, providing evidence that the partially compensated RMR in the warm hypercapnic fish was exclusively induced by temperature and not by elevated PCO_2_.

Following long-term acclimation of *N. rossii* to higher temperatures and *P*CO_2_, animal condition displayed pronounced changes. While control values of HSI and condition factor were within the same range reported recently for *N. rossii* caught in Potter Cove, King George Island [28], they were reduced in warm and hypercapnia acclimated *N. rossii*, although the fish were fed to satiation (control HSI 1.83, condition factor 1.69, warm hypercapnic HSI 0.81, condition factor 1.5; see Table 2). This reduction may be attributed to a reduced aerobic scope caused by elevated RMR (see above) at the high acclimation temperatures chosen.

The fact that the warm-acclimated fish could not completely compensate their RMR to a level comparable to the control animals could indicate beginning limitations in the circulatory system of *N. rossii* and in oxygen supply to tissues*.* As a result, the aerobic scope for the SDA response (specific dynamic action) [70,71] might be limited at warmer temperatures. Consequently, fish may not be capable to ingest sufficient food over time to meet the required energy demand and to sustain basal metabolic rate, even if fed *ad libitum*. To maintain RMR elevated in warmer water, energy stores such as liver fat may be mobilized [72], resulting in the observed lower HSI and condition factor (see above).

The paradigm that Antarctic fish have limited acclimation capacity because of their thermal specialization has been challenged by several studies reporting compensatory adjustments of whole animal respiration, cardiovascular response and blood viscosity at elevated temperatures [11,16,24,66,73]. Most of these studies focused on the cryo-pelagic fish *P. borchgrevinki* or on several *Trematomus* species. In *N. coriiceps*, the congener of *N. rossii*, an acclimation-induced shift in critical thermal maxima (CT_max_) was observed, but the increase was small compared to the shifts observed in other Antarctic species (e.g. *P. brachycephalum, Gobionotothen gibberifrons, T. pennellii and T. hansoni,* see Bilyk and DeVries [66] for further details), further corroborating our conclusions for *N. rossii*.

In general, the measurement of RMR provides a suitable indicator of a species’ thermal tolerance, as limits in oxygen consumption can reflect the onset of whole animal oxygen limitation and associated limitations in circulatory capacity [74]. Nevertheless, the precise determination of aerobic limits under increased temperature and *P*CO_2_ benefits from the combined study of several indicators including enzyme and mitochondrial capacities, the limiting factors in ATP supply.

The mitochondrial state III respiration of the control group rose continuously with rising experimental assay temperatures of 0, 6 and 12°C. The RCR values were stable between 0 and 12°C (see Table 3), indicating efficient mitochondrial coupling up to 12°C. A decrease in Q_10_ from 2.4 (range 0-6°C) to 1.6 (range 6-12°C), indicates that state III respiration became less responsive to temperature at higher assay temperatures, and led to a similar decrease in mitochondrial scope as reported for *N. rossii*[28] and *Lepidonotothen nudifrons*[12] beyond 9°C.

In contrast to the elevation of RMR in the warm normocapnia acclimated *N. rossii*, maximum mitochondrial respiration rates were not significantly higher than in the control group (Figure 2). This was reflected in similar values of RCR (control: 4.6±0.8, warm normocapnia: 4.0±1.0) and Q_10_ between these two groups over the whole range of assay temperatures (Q_10_ from 0 to 12°C, control: 1.6; warm normocapnia: 1.8) (see Table 3). Only the trend towards a reduced thermal slope of mitochondrial state III respiration of the warm normocapnic fish (linear regression analysis of state III respiration from 0 to 12°C assay temperature; warm normocapnic: slope 0.22 [(nmol O_2_*min^-1^*mg^-1^)*1°C], control group: 0.39) could point towards a beginning compensation of mitochondrial respiration. The partial (type III) compensation at the whole animal level (RMR, Figure 1) could therefore originate at the mitochondrial level, possibly underpinning relevant adjustments of the cardiovascular system.

Interestingly, cold hypercapnia acclimation led to significantly reduced state III respiration at acute assay temperatures of 6°C and 12°C compared to the control group, accompanied by a significantly reduced mean RCR over the whole range of assay temperatures (Table 2). Similarly, state III respiration was depressed in the warm hypercapnia acclimated *N. rossii*, below that of the control group (Figure 2), and showed significantly reduced RCRs, indicating a clear effect of elevated ambient *P*CO_2_ on mitochondrial metabolism. This effect did not translate into a change in whole animal RMR but may reflect a decrease in tissue and whole animal aerobic and functional scope. In contrast, a down-regulation in resting aerobic metabolic rate occurred under acute hypercapnic acidosis in muscle tissue of the invertebrate *Sipunculus nudus*, reflecting a reduction in ATP consuming processes of maintenance metabolism (e.g. anabolic/ catabolic protein metabolism) [75,76]. Such energy savings might also occur in fishes and affect proteins involved in mitochondrial respiration (e.g. reduced citrate synthase activities in hypercapnia acclimated *Sparus aurata*[40]), thereby causing a lower state III respiration. Since state IV respiration remained unchanged after cold or warm hypercapnia acclimation (data not shown), the reduced coupling capacities were likely caused by the reduced state III respiration (per mg mitochondrial protein) and not by increased proton leak rates. The reduced COX activities (per mg cellular protein) (Figure 3) in the liver of both cold and warm hypercapnia acclimated *N. rossii* support this hypothesis and are in line with the projected changes in protein activity, including possible modifications in the mitochondrial membrane.

The differences observed at the mitochondrial level only partially reflected the whole animal level (see Figure 1), specifically in that the mitochondrial studies exclusively concentrated on liver tissue, which only constitutes a fraction of whole animal metabolism. A possible whole organism consequence of such capacity limits in mitochondrial metabolism under conditions of elevated energy demand (e.g. activity, reproduction) may be shifts in metabolic pathways [40] and a decrease in aerobic scope under long-term elevated *P*CO_2_. Further alterations may include a reduction in growth or behavioural capacities under long-term increased *P*CO_2_, as observed in coral reef fish (*Amphiprion percula* &*Neopomacentrus azysron*) [77-79].

### Acid–base regulation

The changes in mitochondrial capacities may be related to shifts in extra- and particularly intracellular acid–base status. The liver pH_i_ in control *N. rossii* of this study (pH_i_ 7.08, Table 4) were similar to values recorded for the eelpout *Z. viviparus* (pH_i_ 7.06, [63]). The pH_i_ values of the white muscle samples (e.g. control group; pH_i_ 7.325) were close to values reported for Antarctic and non-Antarctic fish in other studies (e.g. *G. morhua* 7.34, [80]; *P. brachycephalum* 7.42-7.43 [81], *Harpagifer antarcticus* pH_i_ 7.36 at 1°C [49], 7.33 *N. coriiceps*[82]). In the warm normocapnia acclimated group, white muscle values followed the α-stat pattern [83], with a lowering of pH with increasing temperature by −0.014 pH units/°C. Such a rise in body temperature also caused a linear drop of pH_i_ in white muscle of the North Sea eelpout *Z. viviparus* (−0.016 pH units/°C) [63]. The illustration of intracellular acid–base parameters in the pH-bicarbonate diagram (Figure 4) emphasizes a defence of liver pH_i_ by the non-bicarbonate buffer system (such as proteins or amino acid residues) in the cold hypercapnia acclimated fish, in similar ways as recorded e.g. for *G. morhua*[80] or freshwater catfish *Liposarcus pardalis*[43].

In the liver of the warm hypercapnia acclimated *N. rossii* (Figure 4), pH_i_ was compensated by intracellular HCO_3_^-^ accumulation, in parallel to the findings in the blood (pH_e_) and muscle pH_i_. This compensation of chronically increased *P*CO_2_ of both cold and warm groups may have contributed to the observed shifts in metabolic steady state towards slightly alkaline pH values. The long-term reaction to acute changes in acid–base status may include shifts in the use of metabolic substrates by favouring oxidative decarboxylation of dicarboxylic acids (malate, glutamate/ aspartate) [75,84]. These reactions could help to reduce the elevated proton load under chronic elevated *P*CO_2_, thereby playing an important role in the buffering of changes in the acid–base status. Nevertheless, such modifications appear to be insufficient to maintain full mitochondrial capacities in *N. rossii*, paralleled by the observed reduced COX activities and RCR of the cold/ warm hypercapnic mitochondria.

During long-term elevated ambient *P*CO_2_, CO_2_ enters the mitochondria by diffusion, yielding an increase in proton and [HCO_3_^-^ levels. Taking into account the pH and total CO_2_ gradient maintained between mitochondria and the cytoplasm under control conditions, liver mitochondrial [HCO_3_^-^ of the warm hypercapnic animals were up to 10 mmol/l higher than in the warm normocapnic group (or 4 mmol/l in the cold hypercapnic group compared to their controls); calculated after [47,85]. Earlier studies of the acute effects of alkaline pH and increased [HCO_3_^-^ on liver mitochondria revealed inhibitions in the TCA-cycle [86], thereby lowering mitochondrial respiratory capacities and their capacity to supply ATP. In trout (*O. mykiss*) hepatocytes, acutely increased [HCO_3_^-^ at 1 kPa *P*CO_2_ also depresses mitochondrial metabolism via interruptions in the TCA-cycle, possibly caused by alterations in citrate and phosphate transport [87].

Intracellular acid–base regulation is supported by the respective adjustments in extracellular acid–base status, e.g. the accumulation of extracellular bicarbonate during compensation for the respiratory acidosis [88]. The same shift in ‘set points’ towards alkaline values observed at the intracellular level occurred in the blood. In contrast to other pH_e_ values recorded for temperate marine fish (e.g. cod 7.95 [80], flounder 7.78 [89], seabream 7.65 [40]), the extracellular pH of *N. rossii* was quite low in the present study (pH 7.44 in the control group). A study by Egginton [90] revealed a low blood pH of 7.5 for *N. coriiceps* directly after capture, which increased to 7.7 over 96 hours during recovery from landing stress. In cannulated *N. coriiceps* pH_e_ increased from 7.5 to 8.0 during recovery, a value consistent with the blood pH of 8.01 measured for *N. rossii* by Egginton et al. [64]. pH values measured in the present study may be lower than these values due to ‘grab and stab’ effects, as the cannulation of animals was experimentally not possibly. Nevertheless, these handling effects should have affected measurements in all experimental groups in similar ways and thereby still allow for comparison between the different acclimation groups.

As expected for marine teleost fish (e.g. *Conger conger*[91], *G. morhua*[80] or *Sparus aurata*[40]), the acute acidosis evoked by higher environmental *P*CO_2_ was compensated for by a significant increase in plasma [HCO_3_^-^ in both the cold and warm hypercapnic groups. The depiction in the pH-bicarbonate diagram (Figure 5) shows that the increase in plasma [HCO_3_^-^ cannot solely be attributed to extracellular non-bicarbonate buffering. Instead, combined acid–base parameters were positioned above the non-bicarbonate buffer line, likely due to the involvement of proton equivalent ion transfer processes [92]. Although the pattern of compensation is similar for many teleost fish, the [HCO_3_^-^ reached differ between species: e.g. levels reached 22 mM in *C. conger*[91], and 32 mM in cod, respectively [80] when exposed to 1 kPa CO_2_. The exposure to a moderate *P*CO_2_ of 0.2 kPa led to lower but still significantly elevated [HCO_3_^-^ of 11.3 mM in cold hypercapnic *N. rossii.*

The compensation of higher ambient *P*CO_2_ via elevated [HCO_3_^-^_e_ and [HCO_3_^-^_i_ can lead to an increase in ATP demand for ion exchanging processes to maintain [HCO_3_^-^ at this higher level, as it was reported for long-term hypercapnia acclimated eelpout (*Z. viviparus*) [93]. The reaction to this constantly higher ATP demand could be a shift in energy budget with reduced ATP consuming processes, e.g. protein turnover or anabolism [93]. This new metabolic equilibrium under increased metabolic demands for acid–base regulation could result in shifted ‘set points’, as we observed in the warm or cold hypercapnia acclimated *N. rossii*, with pH shifted towards alkaline values and thus a constant, slight metabolic alkalosis in both groups.

Both temperature and hypercapnia influence blood parameters in Antarctic fish, which has been demonstrated for blood osmolarity after thermal acclimation in notothenioids [94]. This explains the observed decreased serum osmolarities in our warm acclimated animals. The unaffected osmolarities in cold hypercapnia acclimated *N. rossii* are in line with earlier findings by Larsen et al. [80] for cod (*G. morhua*) exposed to 1 kPa CO_2_. Although we observed hypercapnia induced changes in ion regulation, the higher [HCO_3_^-^_e_ in the blood are too small to significantly alter total osmolarity. Hence, the changes in osmolarity can exclusively be attributed to long-term warm acclimation.

The haematocrit levels of *N. rossii* were unaffected by warm and/ or hypercapnia acclimation, and within the range reported for its sympatric sister species *N. coriiceps*[26,95] (Table 2). While acute warming causes an elevation of haematocrit in red-blooded notothenioids [26], long-term warm acclimation leaves haematocrit levels constant, consistent with results from other studies on Antarctic notothenioids [94]. Thus, the oxygen carrying capacities of the blood of warm and/ or hypercapnia acclimated *N. rossii* do not seem to be limiting under these conditions.

It has been assumed that the extracellular non-bicarbonate buffering is mostly accomplished by proteins in the blood [96], and thus strongly depends on haematocrit [97], which varies greatly between fish species, also among Antarctic fish species [26,28,98,99]. The haematocrit levels measured in *N. rossii* (28–31, see Table 2) thus result in high blood β_NB_ values (30.3 mmol/l pH). Similarly, the red-blooded Antarctic fish *Dissostichus mawsoni* and *P. borchgrevinki* showed higher β_NB_ values (~28 and 18 mmol/l pH, respectively) than the haemoglobinless icefish *Pagetopsis macropterus* (β_NB_ ~3 mmol/l pH) [100].

Some possible limitations in oxygen availability may have occurred at the intracellular level in the warm normocapnia acclimated fish, where liver pH_i_ was lower than in the control group. This pH difference cannot be exclusively attributed to α-stat regulation [83], as pH_i_ changed by −0.032 pH units/°C. Possibly, the high acclimation temperature of 7°C led to limiting oxygen supply to the liver tissue as a consequence of elevated metabolic demand,, resulting in a slight contribution of anaerobic metabolism and thereby lactate production, thus shifting the pH_i_ of the warm normocapnic group to acidic values. Nevertheless, other tissues with lower metabolic loads than liver, such as white muscle, may still be able to metabolise anaerobic end products to some degree, allowing the animals to survive at these warmer temperatures (4–6 weeks acclimation time in this study).

We did, however, not observe elevated lactate values in the blood of warm normocapnic/ hypercapnic fish, and the generally low lactate values of *N. rossii* were similar to those measured in *N. rossii* and *N. coriiceps* earlier [28,101]. Only in the cold hypercapnia acclimated animals, lactate levels were slightly elevated, but are likely the result of minor handling stress and do not originate from a beginning anaerobic metabolism in the liver, as they are still close to the levels of 1 mM reported for *N. coriiceps* under natural conditions [26,90].

A higher ATP demand under conditions of elevated temperature in combination with an intracellular acidosis might shift or even impair liver functionality over a longer time-scale in the warm-acclimated animals (normocapnia/ hypercapnia), which could relate to the reduced HSI in the animals of the present study (see Table 2).

## Conclusion

This study investigated the thermal plasticity and acclimation abilities to higher temperature and *P*CO_2_ levels of the Antarctic teleost fish *N. rossii*, by studying metabolic responses at different organisation levels (whole animal, blood, cellular and mitochondrial level).

At the whole animal level, our findings reveal partial compensation of RMR in the long-term warm normocapnia and hypercapnia acclimated fish in comparison to acute warm (7°C) exposed *N. rossii*. Long-term acclimation to 0.2 kPa CO_2_ had no effect on RMR.

In the mitochondria, we observed only limited compensation of state III respiration following normocapnic warm acclimation. In contrast, both warm and cold hypercapnia acclimation led to reduced mitochondrial capacities, possibly mediated by changes in the TCA-cycle or the whole mitochondria, as indicated by reduced enzyme capacities.

In cold and warm hypercapnia acclimated fish, we observed shifts in the ‘set points’ of acid–base regulation to more alkaline values at both extra- and intracellular levels, mediated by actively accumulated [HCO_3_^-^]. These shifts may be involved in the hypercapnia-induced changes in cellular and mitochondrial energy demand. During long-term hypercapnia, shifts towards oxidative decarboxylation processes may maintain new acid–base equilibria. As the reduced mitochondrial capacities of the cold and warm hypercapnia acclimated fish were not visible in whole animal respiration, *N. rossii* might be limited in energy supply and aerobic scope for e.g. activity, growth and reproduction.

In the context of other data available for other high-Antarctic notothenioids [16,73], our data suggest that among the notothenioids the cold-adapted *N. rossii* will have only a moderate scope for acclimation and tolerance towards ocean acidification and warming of the Southern Ocean. At the chosen temperature of warm acclimation (7°C), the liver function of *N. rossii* may shift or become disturbed, thereby likely reducing whole animal performance over longer time-scales.

## Competing interests

The authors declare that they have no competing interests.

## Authors’ contributions

AS and FCM designed the study, carried out the animal capture, acclimation, mitochondrial respiration experiments and extracellular acid–base determination and drafted the manuscript. AS performed the data analyses and interpretation. SB carried out the intracellular acid–base determination and contributed to writing the manuscript. EL performed the enzyme measurements. KM participated in the coordination of the study and contributed to revising the manuscript. HOP participated in the study design and substantially contributed to writing the manuscript. All authors read and approved the final version of the manuscript.

## References

[B1] Ferron FA Simones JC Aquino FE Setzer AW Air temperature series for King George Island, Antarcitca Pesquisa Ant Brasil 2004 4 155 169

[B2] Cook AJ Fox AJ Vaughan DG Ferrigno JG Retreating Glacier Fronts on the Antarctic Peninsula over the Past Half-Century Science 2005 308 541 544 10.1126/science.1104235 15845851

[B3] Turner J Colwell SR Marshall GJ Lachlan-Cope TA Carleton AM Jones PD Lagun V Reid PA Iagovkina S Antarctic climate change during the last 50 years Int J Climatol 2005 25 279 294 10.1002/joc.1130

[B4] Meredith PM King CJ Rapid climate change in the ocean west of the Antarctic Peninsula during the second half of the 20th century 2005 Washington, DC, USA: American Geophysical Union

[B5] Turner J Lachlan-Cope TA Colwell S Marshall GJ Connolley WM Significant Warming of the Antarctic Winter Troposphere Science 2006 311 1914 1917 10.1126/science.1121652 16574865

[B6] Clarke A Murphy EJ Meredith MP King JC Peck LS Barnes DKA Smith RC Climate change and the marine ecosystem of the western Antarctic Peninsula Philos Trans R Soc Lond B Biol Sci 2007 362 149 166 10.1098/rstb.2006.1958 17405211 PMC1764833

[B7] Sabine CL Feely RA Gruber N Key RM Lee K Bullister JL Wanninkhof R Wong CS Wallace DW Tilbrook B The oceanic sink for anthropogenic CO2 Science 2004 305 367 371 10.1126/science.1097403 15256665

[B8] Caldeira K Wickett ME Anthropogenic carbon and ocean pH Nature 2003 425 365 10.1038/425365a 14508477

[B9] Caldeira K Wickett ME Ocean model predictions of chemistry changes from carbon dioxide emissions to the atmosphere and ocean: The ocean in a high-CO2 world 2005 Washington, DC, USA: American Geophysical Union

[B10] Cao L Caldeira K Atmospheric CO2 stabilization and ocean acidification Geophys Res Lett 2008 35 L19609

[B11] Somero GN DeVries AL Temperature tolerance of some Antarctic fishes Science 1967 156 257 258 10.1126/science.156.3772.257 6021046

[B12] Hardewig I Peck LS Pörtner HO Thermal Sensitivity of Mitochondrial Function in the Antarctic Notothenioid Lepidonotothen nudifrons J Comp Physiol B 1999 169 597 604 10.1007/s003600050260

[B13] Mark FC Bock C Pörtner HO Oxygen-limited thermal tolerance in Antarctic fish investigated by MRI and 31P-MRS Am J Physiol Regul Integr Comp Physiol 2002 283 R1254 R1262 12376420 10.1152/ajpregu.00167.2002

[B14] Peck LS Prospects for survival in the Southern Ocean: vulnerability of benthic species to temperature change Antarct Sci 2005 17 497 10.1017/S0954102005002920

[B15] Verde C Parisi E di Prisco G The evolution of thermal adaptation in polar fish Gene 2006 385 137 145 16757135 10.1016/j.gene.2006.04.006

[B16] Franklin CE Davison W Seebacher F Antarctic fish can compensate for rising temperatures: thermal acclimation of cardiac performance in Pagothenia borchgrevinki J Exp Biol 2007 210 3068 3074 10.1242/jeb.003137 17704081

[B17] Pörtner HO Climate-dependent evolution of Antarctic ectotherms: An integrative analysis Deep Sea Res Part 2 Top Stud Oceanogr 2006 53 1071 1104 10.1016/j.dsr2.2006.02.015

[B18] Pörtner HO Climate change and temperature-dependent biogeography: oxygen limitation of thermal tolerance in animals Naturwissenschaften 2001 88 137 146 10.1007/s001140100216 11480701

[B19] Mark FC Hirse T Pörtner HO Thermal sensitivity of cellular energy budgets in some Antarctic fish hepatocytes Polar Biol 2005 28 805 814 10.1007/s00300-005-0018-0

[B20] Clarke A What is cold adaptation and how should we measure it? Amer Zool 1991 31 81 92

[B21] Peck LS Prospects for survival in the Southern Ocean: vulnerability of benthic species to temperature change Antarctic Science-Institutional Subscription 2005 17 497 508

[B22] Pörtner HO Lannig G Richards JG, Farrell AP, Brauner C Oxygen and capacity limited thermal tolerance Fish Physiology, Vol 27: Hypoxia 2009 Burlington: Academic 143 191

[B23] Lowe CJ Davison W Thermal sensitivity of scope for activity in Pagothenia borchgrevinki, a cryopelagic Antarctic nototheniid fish Polar Biol 2006 29 971 977 10.1007/s00300-006-0139-0

[B24] Seebacher F Davison W Lowe CJ Franklin CE A falsification of the thermal specialization paradigm: compensation for elevated temperatures in Antarctic fishes Biol Lett 2005 1 151 154 10.1098/rsbl.2004.0280 17148152 PMC1626235

[B25] Podrabsky J Somero G Inducible heat tolerance in Antarctic notothenioid fishes Polar Biol 2006 30 39 43 10.1007/s00300-006-0157-y

[B26] Beers JM Sidell BD Thermal tolerance of Antarctic notothenioid fishes correlates with level of circulating hemoglobin Physiol Biochem Zool 2011 84 353 362 10.1086/660191 21743249

[B27] Pörtner HO Lucassen M Storch D Farrell AP, Steffensen JF Metabolic biochemistry: its role in thermal tolerance and in the capacities of physiological and ecological function The Physiology of Polar Fishes. Volume 21 2005 San Diego: Elsevier Academic Press 79 154 [Hoar WS, Randall DR, Farrell AP (Series Editor): Fish Physiology]

[B28] Mark FC Lucassen M Strobel A Barrera-Oro E Koschnick N Zane L Patarnello T Pörtner HO Papetti C Mitochondrial Function in Antarctic Nototheniids with ND6 Translocation PLoS One 2012 7 e31860 10.1371/journal.pone.0031860 22363756 PMC3283701

[B29] Pörtner HO Hardewig I Peck LS Mitochondrial Function and Critical Temperature in the Antarctic Bivalve, Laternula elliptica Comp Biochem Physiol A 1999 124 179 189 10.1016/S1095-6433(99)00105-1

[B30] Heise K Puntarulo S Pörtner HO Abele D Production of reactive oxygen species by isolated mitochondria of the Antarctic bivalve Laternula elliptica (King and Broderip) under heat stress Comp Biochem Physiol C Toxicol Pharmacol 2003 134 79 90 10.1016/S1532-0456(02)00212-0 12524020

[B31] Johnston I Guderley H Franklin C Crockford T Kamunde C Are mitochondria subject to evolutionary temperature adaptation? J Exp Biol 1994 195 293 9317834 10.1242/jeb.195.1.293

[B32] Urschel M O’Brien K Mitochondrial function in Antarctic notothenioid fishes that differ in the expression of oxygen-binding proteins Polar Biol 2009 32 1323 1330 10.1007/s00300-009-0629-y

[B33] Lemieux H Tardif J Dutil J Blier P Thermal sensitivity of cardiac mitochondrial metabolism in an ectothermic species from a cold environment, Atlantic wolffish (Anarhichas lupus) J Exp Mar Bio Ecol 2010 384 113 118 10.1016/j.jembe.2009.12.007

[B34] Walther K Sartoris F Bock C Pörtner HO Impact of anthropogenic ocean acidification on thermal tolerance of the spider crab Hyas araneus Biogeosciences Discussions 2009 6 2837 2861 10.5194/bgd-6-2837-2009

[B35] Pörtner HO Farrell AP Ecology. Physiology and climate change Science 2008 322 690 692 10.1126/science.1163156 18974339

[B36] Metzger R Sartoris F Langenbuch M Pörtner HO Influence of elevated CO2 concentrations on thermal tolerance of the edible crab Cancer pagurus J Therm Biol 2007 32 144 151 10.1016/j.jtherbio.2007.01.010

[B37] Foss A Rųsnes B Ųiestad V Graded environmental hypercapnia in juvenile spotted wolffish (Anarhichas minor Olafsen): effects on growth, food conversion efficiency and nephrocalcinosis Aquaculture 2003 220 607 617 10.1016/S0044-8486(02)00613-0

[B38] Fivelstad S Olsen AB Asgård T Baeverfjord G Rasmussen T Vindheim T Stefansson S Long-term sublethal effects of carbon dioxide on Atlantic salmon smolts (Salmo salar L.): ion regulation, haematology, element composition, nephrocalcinosis and growth parameters Aquaculture 2003 215 301 319 10.1016/S0044-8486(02)00048-0

[B39] Melzner F Gutowska M Hu M Stumpp M Acid–base regulatory capacity and associated proton extrusion mechanisms in marine invertebrates: An overview Comp Biochem Physiol A Mol Integr Physiol 2009 153 S80

[B40] Michaelidis B Spring A Pörtner HO Effects of long-term acclimation to environmental hypercapnia on extracellular acid–base status and metabolic capacity in Mediterranean fish Sparus aurata Mar Biol 2007 150 1417 1429 10.1007/s00227-006-0436-8

[B41] Pörtner HO Bock C Reipschläger A Modulation of the cost of pHi regulation during metabolic depression: A P-31-NMR study in invertebrate (Sipunculus nudus) isolated muscle J Exp Biol 2000 203 2417 2428 10903156 10.1242/jeb.203.16.2417

[B42] Bock C Sartoris FJ Wittig RM Pörtner HO Temperature-dependent pH regulation in stenothermal Antarctic and eurythermal temperate eelpout (Zoarcidae): an in-vivo NMR study Polar Biol 2001 24 869 874 10.1007/s003000100298

[B43] Brauner CJ Wang T Wang Y Richards JG Gonzalez RJ Bernier NJ Xi W Patrick M Val AL Limited extracellular but complete intracellular acid–base regulation during short-term environmental hypercapnia in the armoured catfish, Liposarcus pardalis J Exp Biol 2004 207 3381 3390 10.1242/jeb.01144 15326214

[B44] Lannig G Eilers S Pörtner HO Sokolova IM Bock C Impact of ocean acidification on energy metabolism of oyster, Crassostrea gigas - changes in metabolic pathways and thermal response Mar Drugs 2010 8 2318 2339 10.3390/md8082318 20948910 PMC2953406

[B45] Abe H Role of histidine-related compounds as intracellular proton buffering constituents in vertebrate muscle Biochemistry c/c of Biokhimiya 2000 65 757 765 10951092

[B46] Sartoris FJ Bock C Pörtner HO Temperature-dependent pH regulation in eurythermal and stenothermal marine fish: an interspecies comparison using P-31-NMR J Therm Biol 2003 28 363 371 10.1016/S0306-4565(03)00012-3

[B47] Pörtner HO Determination of Intracellular Buffer Values After Metabolic Inhibition by Fluoride and Nitrilotriacetic Acid Respir Physiol 1990 81 275 288 10.1016/0034-5687(90)90051-Y 2124718

[B48] Young S Egginton S Allometry of skeletal muscle fine structure allows maintenance of aerobic capacity during ontogenetic growth J Exp Biol 2009 212 3564 3575 10.1242/jeb.029512 19837898

[B49] Moerland TS Egginton S Intracellular pH of muscle and temperature: insight from in vivo 31P NMR measurements in a stenothermal Antarctic teleost (Harpagifer antarcticus) J Therm Biol 1998 23 275 282 10.1016/S0306-4565(98)00016-3

[B50] Sosinski JSK Biomass estimate of commercial fish on the shelf of South Georgia by the swept area method Bull Sea Fish Inst 1987 5-6 8 14

[B51] Duhamel G Distribution and abundance of fish on the Kerguelen Island shelf 1987 1985 Stockholm: Proc V Congr europ Ichthyol

[B52] Casaux R Mazzotta A Barrera-Oro E Seasonal aspects of the biology and diet of nearshore nototheniid fish at Potter Cove, South Shetland Islands, Antarctica Polar Biol 1990 11 63 72

[B53] Gon O Heemstra P Fishes of the Southern Ocean 1990 Grahamstown, South Africa: J.L.B. Smith Institute for Ichthyology

[B54] Everson I Inshore fishes from the South Orkney and South Shetland Islands, the Antarctic Peninsula and South Georgia Br Ant Surv Bull 1969 19 89 96

[B55] Schloss I Ferreyra GA González O Atencio A Fuentes V Tosonotto G Mercuri G Sahade R Tatián M Abele D Potter C, Wiencke C, Ferreyra G, Abele D, Marenssi S Long term hydrographic conditions and climate trends The Potter Cove coastal ecosystem, Antarctica Synopsis of research performed 1999–2006 at the Dallmann Laboratory and Jubany Station, King George Island (Isla 25 de Mayo) Ber Polarforsch Meeres 2008

[B56] Caldeira K Wickett M Ocean model predictions of chemistry changes from carbon dioxide emissions to the atmosphere and ocean J Geophys Res 2005 110 1 12

[B57] Pierrot D Lewis E Wallace DWR MS Excel Program Developed for CO2 System Calculations Book MS Excel Program Developed for CO2 System Calculations 2006 ORNL/CDIAC-105a edition City: Carbon Dioxide Information Analysis Center, Oak Ridge National Laboratory, U.S. Department of Energy

[B58] Johnston IA Clarke A Ward P Temperature and metabolic rate in sedentary fish from the Antarctic, North Sea and Indo-West Pacific Ocean Mar Biol 1991 109 191 195 10.1007/BF01319386

[B59] Ricker WE Computation and interpretation of the biological statistics of fish populations Bull Fish Res Board Can 1975 191 382

[B60] Bradford M A rapid and sensitive method for the quantitation of microgram quantities of protein utilizing the principle of protein-dye binding Anal Biochem 1976 72 248 254 10.1016/0003-2697(76)90527-3 942051

[B61] Moyes C Mathieu-Costello O Tsuchiya N Filburn C Hansford R Mitochondrial biogenesis during cellular differentiation Am J Physiol Cell Physiol 1997 272 C1345 10.1152/ajpcell.1997.272.4.C1345 9142861

[B62] Heisler N Acid–base Regulation in Animals 1986 Amsterdam: Elsevier Science Publisher B.V. (Biomedical Division)

[B63] Van Dijk PL Hardewig I Pörtner HO Temperature-dependent shift of pHi in fish white muscle: contributions of passive and active processes Am J Physiol 1997 272 R84 R89 9038994 10.1152/ajpregu.1997.272.1.R84

[B64] Egginton S Taylor E Wilson R Johnston I Moon T Stress response in the Antarctic teleosts (Notothenia neglecta Nybelin and N. rossii Richardson) J Fish Biol 1991 38 225 235 10.1111/j.1095-8649.1991.tb03108.x

[B65] Noack S Statistische Auswertung von Menü-und Versuchsdaten mit Taschenrechner und Tischcomputer 1980 Berlin-New York: De Gruyter

[B66] Bilyk KT DeVries AL Heat tolerance and its plasticity in Antarctic fishes Comp Biochem Physiol A Mol Integr Physiol 2011 158 382 390 10.1016/j.cbpa.2010.12.010 21159323

[B67] Vanella FA Calvo J Influence of temperature, habitat and body mass on routine metabolic rates of Subantarctic teleosts Sci Mar 2005 69 317 323

[B68] Pörtner HO Mark FC Bock C Oxygen limited thermal tolerance in fish? Answers obtained by nuclear magnetic resonance techniques Respir Physiol Neurobiol 2004 141 243 260 10.1016/j.resp.2004.03.011 15288597

[B69] Precht H Christophersen J Hensel H Larcher W Temperature and life 1973 Berlin, Heidelberg: Springer

[B70] Fry F The Effect of Environmental Factors on the Physiology of Fish Fish Physiol 1971 6 1 98

[B71] Jobling M The influences of feeding on the metabolic rate of fishes: a short review J Fish Biol 1981 18 385 400 10.1111/j.1095-8649.1981.tb03780.x

[B72] Lannig G Storch D Pörtner HO Aerobic mitochondrial capacities in Antarctic and temperate eelpout (Zoarcidae) subjected to warm versus cold acclimation Polar Biol 2005 28 575 584 10.1007/s00300-005-0730-9

[B73] Robinson E Davison W The Antarctic notothenioid fish Pagothenia borchgrevinki is thermally flexible: acclimation changes oxygen consumption Polar Biol 2008 31 317 326 10.1007/s00300-007-0361-4

[B74] Melzner F Bock C Pörtner HO Temperature-dependent oxygen extraction from the ventilatory current and the costs of ventilation in the cephalopod Sepia officinalis J Comp Physiol B 2006 176 607 621 10.1007/s00360-006-0084-9 16710699

[B75] Langenbuch M Pörtner HO Changes in metabolic rate and N excretion in the marine invertebrate Sipunculus nudus under conditions of environmental hypercapnia: identifying effective acid–base variables J Exp Biol 2002 205 1153 1160 11919274 10.1242/jeb.205.8.1153

[B76] Langenbuch M Bock C Leibfritz D Pörtner HO Effects of environmental hypercapnia on animal physiology: A C-13 NMR study of protein synthesis rates in the marine invertebrate Sipunculus nudus Comp Biochem Physiol A Mol Integr Physiol 2006 144 479 484 10.1016/j.cbpa.2006.04.017 16753322

[B77] Munday PL Dixson DL McCormick MI Meekan M Ferrari MC Chivers DP Replenishment of fish populations is threatened by ocean acidification Proc Natl Acad Sci U S A 2010 107 12930 12934 10.1073/pnas.1004519107 20615968 PMC2919925

[B78] Domenici P Allan B McCormick MI Munday PL Elevated carbon dioxide affects behavioural lateralization in a coral reef fish Biol Lett 2012 8 78 81 10.1098/rsbl.2011.0591 21849307 PMC3259963

[B79] Nilsson GE Dixson DL Domenici P McCormick MI Sørensen C Watson SA Munday PL Near-future carbon dioxide levels alter fish behaviour by interfering with neurotransmitter function Nat Clim Chang 2012 2 3 201 204 10.1038/nclimate1352

[B80] Larsen BK Pörtner HO Jensen FB Extra- and Intracellular Acid–base Balance and Ionic Regulation in cod (Gadus Morhua) during Combined and Isolated Exposures to Hypercapnia and Copper Mar Biol 1997 128 337 346 10.1007/s002270050099

[B81] Van Dijk PLM Tesch C Hardewig I Pörtner HO Physiological disturbances at critically high temperatures: A comparison between stenothermal Antarctic and eurythermal temperate eelpouts (Zoarcidae) J Exp Biol 1999 202 3611 3621 10574738 10.1242/jeb.202.24.3611

[B82] Taylor S Egginton S Taylor E Franklin C Johnston I Estimation of intracellular pH in muscle of fishes from different thermal environments J Therm Biol 1999 24 199 208 10.1016/S0306-4565(99)00013-3

[B83] Reeves RB An imidazole alphastat hypothesis for vertebrate acid–base regulation: tissue carbon dioxide content and body temperature in bullfrogs Respir Physiol 1972 14 219 236 10.1016/0034-5687(72)90030-8 4537783

[B84] Pörtner HO Contributions of anaerobic metabolism to pH regulation in animal tissues: theory J Exp Biol 1987 131 69 87 3694119 10.1242/jeb.131.1.69

[B85] Pörtner HO MacLatchy LM Toews DP Acid–base regulation in the toad Bufo marinus during environmental hypoxia Respir Physiol 1991 85 217 230 10.1016/0034-5687(91)90063-O 1658901

[B86] Simpson DP Regulation of renal citrate metabolism by bicarbonate ion and pH: observations in tissue slices and mitochondria J Clin Invest 1967 46 225 10.1172/JCI105525 6018760 PMC297041

[B87] Walsh PJ Mommsen TP Moon T Perry S Effects of acid–base variables on in vitro hepatic metabolism in rainbow trout J Exp Biol 1988 135 231 241 3131477 10.1242/jeb.135.1.231

[B88] Pörtner HO Reipschläger A Heisler N Acid–base Regulation, Metabolism and Energetics in Sipunculus nudus as a Function of Ambient Carbon Dioxide Level J Exp Biol 1998 201 43 55 9390935 10.1242/jeb.201.1.43

[B89] Nonnotte G Truchot JP Time course of extracellular acid–base adjustments under hypo- or hyperosmotic conditions in the euryhaline fish Platichthys flesus J Fish Biol 1990 36 181 190 10.1111/j.1095-8649.1990.tb05594.x

[B90] Egginton S Stress response in two Antarctic teleosts (Notothenia coriiceps Richardson and Chaenocephalus aceratus Lönnberg) following capture and surgery J Comp Physiol B 1994 164 482 491 10.1007/BF00714586

[B91] Toews D Holeton G Heisler N Regulation of the acid–base status during environmental hypercapnia in the marine teleost fish Conger conger J Exp Biol 1983 107 9 20 6668465 10.1242/jeb.107.1.9

[B92] Pörtner HO Ecosystem effects of ocean acidification in times of ocean warming: a physiologist's view Mar Ecol Prog Ser 2008 373 203 217

[B93] Deigweiher K Koschnick N Pörtner HO Lucassen M Acclimation of ion regulatory capacities in gills of marine fish under environmental hypercapnia Am J Physiol Regul Integr Comp Physiol 2008 295 R1660 R1670 10.1152/ajpregu.90403.2008 18799636

[B94] Hudson H Brauer P Scofield M Petzel D Effects of warm acclimation on serum osmolality, cortisol and hematocrit levels in the Antarctic fish, Trematomus bernacchii Polar Biol 2008 31 991 997 10.1007/s00300-008-0438-8

[B95] Heise K Estevez M Puntarulo S Galleano M Nikinmaa M Pörtner HO Abele D Effects of seasonal and latitudinal cold on oxidative stress parameters and activation of hypoxia inducible factor (HIF-1) in zoarcid fish J Comp Physiol B 2007 177 765 777 10.1007/s00360-007-0173-4 17579869

[B96] Cameron JN Acid–base homeostasis: past and present perspectives Physiol Zool 1989 62 845 865

[B97] Wood CM McDonald D McMahon B The influence of experimental anaemia on blood acid–base regulation in vivo and in vitro in the starry flounder (Platichthys stellatus) and the rainbow trout (Salmo gairdneri) J Exp Biol 1982 96 221

[B98] Smit G Hattingh J Burger A Haematological assessment of the effects of the anaesthetic MS 222 in natural and neutralized form in three freshwater fish species: interspecies differences J Fish Biol 1979 15 633 643 10.1111/j.1095-8649.1979.tb03672.x

[B99] Kunzmann A Blood Physiology and Ecological Consequences in Weddell Sea Fishes (Antarctica)PhD 1991

[B100] Wells RMG Respiration of Antarctic fish from McMurdo Sound Comp Biochem Physiol 1987 88 A 417 424 10.1016/0300-9629(87)90056-9 2892614

[B101] Egginton S A comparison of the response to induced exercise in red- and white-blooded Antarctic fishes J Comp Physiol B 1997 167 129 134 10.1007/s003600050056

